# AI-driven drug reposition for pathogens: a new paradigm in pandemic preparedness

**DOI:** 10.3389/fchem.2026.1842082

**Published:** 2026-06-25

**Authors:** Lixuan Ma, Jingshu Zhao, Junwen Luan, Leiliang Zhang

**Affiliations:** 1 Department of Clinical Laboratory Medicine, The First Affiliated Hospital of Shandong First Medical University & Shandong Provincial Qianfoshan Hospital, Jinan, China; 2 Department of Pathogen Biology, School of Clinical and Basic Medical Sciences, Shandong First Medical University & Shandong Academy of Medical Sciences, Jinan, China

**Keywords:** AI-driven, alphafold 3 (AF3), deep integrated network analysis (DINA), drug repositioning, infectious diseases

## Abstract

Faced with a severe outbreak of diseases caused by newly emerging and recurrent pathogens, the development cycle of traditional drugs is long, making it difficult to meet emergency needs. Drug repositioning has become a key strategy for rapidly providing therapies by exploring new therapeutic uses of approved drugs. However, traditional reposition methods have bottlenecks such as slow speed and strong randomness. Artificial intelligence (AI) is revolutionizing drug reposition by analyzing and integrating multi-source data with computational models, dramatically accelerating the discovery process. This article summarizes the core technological approaches of AI-driven drug reposition, including predictions based on network medicine, virtual screening through deep learning models, and association discovery via real-world data mining. Multiple successful cases are presented to verify their effectiveness. Although there are still challenges in terms of data quality, model interpretability, and clinical translation, AI will undoubtedly reshape our drug development paradigm for addressing future public health crises, serving as a pivotal engine for rapid response and precise intervention.

## 1 Introduction: Why do we need AI to reposition anti-infective drugs?

Pathogens, including viruses, bacteria, fungi, parasites, and other diseases caused by infecting hosts, often have a certain infectivity or dissemination potential, and are prone to causing new and recurrent pandemics. According to statistics, pathogenic infectious diseases has become a serious challenge to global public health, with fast spread speed, geographical scope, and socio-economic impact far exceeding the carrying capacity of traditional prevention and control systems. As of 12 August 2024, the outbreak of Monkeypox virus (MPXV) has affected 116 countries, regions and territories, with 99,176 confirmed cases and 208 deaths, resulting in a case mortality rate (CFR) of 0.21% (Otieno et al., 2025). As of August 2025, there have been over 317,000 reported cases of the Chikungunya virus (CHIKV) globally, mainly in the Americas, Africa and Asia (Tee et al., 2025; Wang et al., 2025). Between March 2012 and May 2025, the World Health Organization (WHO) has reported a total of 2,626 laboratory confirmed human cases of MERS-CoV, the Middle East respiratory syndrome coronavirus, with a mortality rate as high as 36.1% (Barry, 2025).

However, diseases often erupt suddenly and spread rapidly, creating sharp contradictions with the long cycles required for traditional drug development. On this basis, as early as the late 1990s, terms such as “Drug Repositioning” and “Drug Repurposing” began to frequently appear in scientific literature and industry reports, and subsequent drug repositioning gradually transitioned from accidental discovery to stable drug research (Rao et al., 2022). Drug repositioning, that is, the use of a drug in an indication other than the one for which it was initially marketed, is a growing trend (Jourdan et al., 2020). For example, the antidepressant Paroxetine has shown potential cardioprotective effects, surpassing its established role as a selective serotonin reuptake inhibitor (SSRI) (Kosić et al., 2025). Thalidomide, a drug withdrawn from the market due to its severe teratogenic effects, has been repurposed for the treatment of multiple myeloma (MM) (Chen et al., 2023). Furthermore, its potential efficacy against Severe Acute Respiratory Syndrome Coronavirus 2 (SARS-CoV-2) infection has been demonstrated by Laura Monteonofrio et al. (Monteonofrio et al., 2024). Leveraging known pharmacokinetic and safety profiles, existing drugs can be repositioned to circumvent early-stage development and significantly accelerate the translation to clinical use (Shahzadi et al., 2025). However, the traditional strategy of using old drugs for new purposes is often hindered by unclear mechanisms, redundant data, and low economic benefits. The prevailing repositioning strategy typically relies on mining large clinical databases or conducting low-throughput, guided laboratory assays. However, this process exhibits significant blindness, randomness, and inefficiency. Consequently, it falls short of systematically exploring the profound and hidden associations between existing drugs and complex diseases.

On the basis of traditional drug development, combined with drug repositioning and AI assisted drug development, the process of drug repositioning discovery can be reversed, guiding a new direction from laboratory to clinical, greatly improving drug development efficiency and reducing drug development costs (Rawat et al., 2024). By integrating multiple omics data and knowledge graph calculations, AI can systematically deconstruct the complex network of ‘drug-target-disease’ (Song et al., 2022), display the three-dimensional (3D) structure of the target protein (Price, 2025) and quickly identify new indications for marketed drugs (Lou et al., 2023). This strategy has demonstrated key value in epidemic response: it significantly shortens the research and development cycle, reduces clinical risks, and ensures drug accessibility. Building an AI-driven drug repositioning platform will become a strategic defense force in response to newly emerging infectious diseases, achieving a fundamental shift from passive response to proactive and precise prevention and control.

This review summarizes three different paradigms for key drug development strategies: traditional drug development, repositioning assisted development, and integrated AI enhanced repositioning pathways. To critically summarize the enhanced drug development strategy of AI + drug repositioning by including successful and failed cases of disease caused by pathogens in various strategies.

## 2 Traditional drug research and development

The traditional drug development process mainly includes i) target research and validation, as well as selecting suitable targets for drug discovery in specific diseases. This process usually involves genetic target evaluation, genomics and proteomics research, and bioinformatics prediction. Typically, target molecule costs $165 million, while target validation requires approximately $205 million, and 75% of the costs are due to failures throughout the process, this process taking 10 years and only 1 in 12 molecules enter clinical trials (Liscano et al., 2020). ii) Screening of lead compounds after target validation, compound adjustment, and confirmation of the optimal compound. Before the emergence of AI, most of them were based on rule-based and empirical models to screen like searching for a needle in a haystack, as well as combinatorial chemistry screening of molecules from molecular libraries (Kate et al., 2023). iii) Preclinical development mainly involved pharmacokinetic studies and toxicity testing of animal models. iii) Apply for clinical trials to conduct clinical studies stage I, II, III, IV, evaluate the efficacy and toxicity of the drug in the target population. The entire process of taking a new chemical entity to the market costs about 2-3 billion US dollars (Adasme et al., 2021) and takes about 15 years (Lou et al., 2023), but the success rate is only 2% (Adasme et al., 2021). To cope with sudden outbreaks of infectious diseases, priority should be given to and efforts should be made to strengthen the early detection, classification, and assessment of the possibility of sustained local transmission of suspected cases (Wu et al., 2020). In the “golden age” of infection control (Chen et al., 2010), vaccines and drugs should be rapidly developed to prevent the virus from spreading out of control. Traditional drug development has shown poor effectiveness in responding to new and recurrent infectious disease outbreaks.

## 3 Drug repositioning

The core of drug repositioning lies in “discovering new uses from what is known”, which can shorten the drug development time to 3–12 years (Hua et al., 2022). In 2004, Ted T. Ashburn and Karl B. Thor defined it as The process of finding new uses outside the scope of the original medical indication for existing drugs (Ashburn and Thor, 2004). Its meaning not only includes modifying the formula (Fernández-García et al., 2025) to improve drug properties or combining therapeutic complementary drugs, but also includes the application of drug targets on new pathogens (Rejinold et al., 2025). N. Rejinold et al. utilized an innovative nano hybrid approach to improve drug properties and enhance the utilization of the insecticide Niclosamide (NIC) for the treatment of monkeypox virus (Rejinold et al., 2025). Raquel Fernández-García et al. used Fixed-dose combination (FDC) technology to create oral compound granules of miltefosine (MLT) and amphotericin B (AmB), solving the oral challenge of AmB for the treatment of visceral leishmaniasis (VL) (Fernández-García et al., 2025). Umesh C Halder discovered that anti HIV drugs Amprenavir, Darunavir, and Saquinavir potentially inhibit MDR-UPEC infection by targeting the active sites of their key enzymes (Halder, 2024). In addition, for the multiple adverse reactions caused by drug side effects, it is also possible to rejuvenate the “old medicine” by combining other drugs to reduce low side effects. Michael T. Sapko et al. found that D-cycloserine (DCS), which is not recommended for neurotoxicity, can be used in combination with Lurasidone for complex urinary tract infections (Sapko et al., 2024). Its main advantage is that it can directly bypass the early stages of traditional research and development. In the face of new or sudden outbreaks of infectious diseases, it combines small molecules from approved databases (such as FDA approved drug libraries) with pathogens and experimentally verifies the inhibitory effect on the critical life cycle of pathogens, greatly improving the success rate of drug development and effectively reducing drug development time. Meanwhile, compared with traditional methods, “old drugs” already have relatively complete pharmacokinetic and safety data (Zheng et al., 2018), mature production processes and supply chain systems, which can reduce the time required for clinical application and subsequent compound commercialization path design by more than 40%. The essence of a separate drug repositioning partner is to provide external innovation dynamic and R&D outsourcing, to achieve rapid commercialization of drugs (Huang, 2020). In summary, low price investment and low time costs greatly enhance the risk resistance of drug development.

## 4 AI^+^optimization for drug repositioning

**FIGURE 1:**
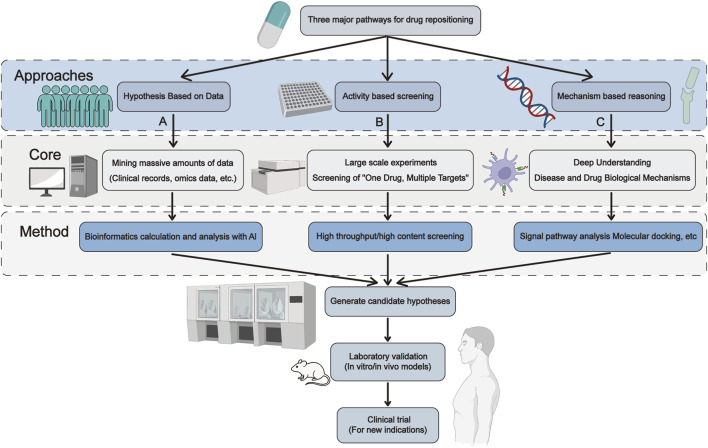
Three main approaches for drug repositioning. The three core paradigms in current drug repositioning research are systematically outlined according to their logical processes and differentiated by approaches, core, and method. **(A)** Hypothesis-based on data utilizes artificial intelligence (AI) to mine clinical records and omics data. **(B)** Activity-based screening employs high-throughput screening technology for large-scale “one drug, multiple targets” experiments. **(C)** Mechanism-based reasoning relies on an in-depth understanding of disease and drug biological mechanisms, performing inference through signaling pathway analysis or molecular docking. The middle section presents the core elements and methods of each approach. Candidate hypotheses generated by the three paths ultimately converge in the process box below, sequentially undergoing essential laboratory verification (in vitro/in vivo models) and clinical trials for new indications. This forms a complete closed loop from initial discovery to clinical translation. Arrows indicate the progressive relationship between research stages.

Drug repositioning is mainly achieved through three approaches: hypothesis based on data, activity based screening, and mechanism based inference (Figure 1).

### 4.1 From massive data through computational analysis/bioinformatics and statistical pattern discovery to hypothesis generation and laboratory validation

Relying on millions of Electronic Health Records (EHR) and real clinical data, researchers have discovered that the incidence rate of another disease unexpectedly decreases in patients who use a certain drug. For example, the most classic drug repositioning case, “acetylsalicylic acid,” was observed by Dr. Lawrence Craven, a general practitioner at Glendale Memorial Hospital in California, who found that aspirin, when used for pain relief in patients undergoing tonsillectomy, also has the side effect of increasing bleeding (Jourdan et al., 2020), which is a classic case of early drug repositioning. Although these findings are often probabilistic, they are also the classic starting point of “from clinical to laboratory” - random accidental discoveries based on large amounts of data. In the 21st century, AI + drug repositioning normalizes rare events and accurately screens target drugs based on real clinical data.

Real world data is being widely applied in epidemiology and pharmacoepidemiology. TriNetX Universal Database (Lee et al., 2023) is a global real-world data and real-world evidence platform that integrates de identified electronic health record from multiple healthcare institutions. Ludwig RJ comprehensively reviewed the methods and applications of using TriNetX database (Ludwig et al., 2025) and summarized relevant cases. However, Hanscheid et al suggested not to systematically explore antimalarial drugs as a treatment for tuberculosis (Hanscheid et al., 2024), which is another success in a sense.

In subsequent omics and biological mechanism research, the mainstream AI assisted tools ABCFold (Elliott et al., 2025) are AlphaFold 3, Boltz-1, and Chai-1 (Table 1).

**TABLE 1:** ABCFold: AlphaFold 3, Boltz-1, and Chai-1.

Project	AlphaFold 3	Boltz-1	Chai-1
Training data	Larger, distillation dataset	Copy AF3 architecture	
Overall performance	Best	Approaching AF3	
Multiple sequence Alignment (MSA)processing method	If running AlphaFold 3with the native JackHMMER search, the resulting MSA will automatically be used for Boltz-1and Chai-1		
	83% of medium predictions maintaining medium DockQ after the ablation	Medium	Medium
Input format and chain tagging method	Maintains the same sequence id mapping as the input JSON, but orders the chains according to label	Mark the input chain in its output with consecutive uppercase letter characters	
Template support	Supports custom templates	Not explicitly mentioned	Kalign needs to be installed
License and commercial use	Not open for commercial use	Fully open-source under MIT license, allowing commercial use	Free for commercial discovery via web interface; python package for non-commercial use
Core technical features	Uses a diffusion network to uniformly predict structures of proteins, DNA, RNA, ligands, etc., with reported ∼50% higher accuracy for some interactions	An open-source model using a diffusion framework	A multimodal model that accepts experimental data as constraints and maintains high performance even in single-sequence mode without MSA
Memorization issue	Found evidence of bias for previously seen structures Performed worse on the post-cutoff structures than the pre-cutoff structures		
	No performance	Trained binding sites predicted more accurately	
Installation method	Manual installation required, model parameters need to be obtained	Can be automatically installed	

ABC Fold refers to a trio of protein structure prediction models—AlphaFold 3, Boltz-1, and Chai-1—that collectively represent the current biomolecular structure modeling. This comparison table evaluates the three models across nine key dimensions.

AlphaFold 3 (AF3) uses Diffusion Network to generate coordinates, which can uniformly predict proteins DNA, RNA, Ligands, and ions, with a 50% higher accuracy than traditional methods, and a success rate of 76% in PoseBusters benchmark testing, demonstrating significant advantages (Abramson et al., 2024). However, due to commercialization barriers of AF3, AlphaFold 2 (AF2) has become a key tool for major basic research, facilitating numerous drug discoveries. The fundamental work in this field has received the highest academic recognition. The Nobel Prize in Chemistry 2024 was divided, one-half awarded to David Baker “for computational protein design”, the other half jointly to Demis Hassabis and John Jumper “for protein structure prediction” (Berger and Eisenberg, 2026); Huixuan Zhao et al. utilized AF2 to conduct virtual screening among FDA-approved drugs, predicting Goserelin as an effective lead compound against SARS-CoV-2 Mpro, and proposed a research plan to combat “X” unknown disease (Zhao et al., 2025). In addition to viruses that have been widely studied and the diseases they cause, Gutnik et al. (2023) also summarized viruses such as rice black-streaked dwarf virus (RBSDV) P10 protein (Liu et al., 2022) and Human roseolovirus (Weaver et al., 2022) which require more research, suggesting the performance of AI in exploring unknown pathogens. In emergency situations where unknown or new variants emerge, AI assistance in drug research to respond to the epidemic is undoubtedly a good method. However, Xin heng et al. evaluated the accuracy of AF3 in predicting ligand-bound G protein-coupled receptors and mentioned that AF3, especially in artificial or composite ligand, is difficult to accurately interact with receptors (He et al., 2025). Combining Tyler M. Rose’s suggestion that the proportion of unmodified drugs from nature in FDA approved drugs is very small and gradually decreasing (Rose et al., 2025). In response to this, the combination of virtual screening and experimental validation is the standard approach for drug discovery, given that modified drugs are currently the mainstream of drug development. Janosch Hennig evaluated the ability of AF3 to predict RNA structure and protein-RNA complexes, and concluded that there is a lack of prediction specifically for non canonical interactions where training data remains scarce (Hennig, 2025).

The Boltz-1 technology path is similar to AF3, using a diffusion model to generate the three-dimensional coordinates of the molecular system. The prediction results are open source, and its code and model weights are fully open under the MIT license, supporting commercial use (Wohlwend et al., 2025). Afterwards, Passaro S et al. also published Boltz-2, a model with experimental method conditioning, distance constraints, and multi-chain template integration for binding affinity prediction The prediction model (Passaro et al., 2025). Chai-1 supports multimodal inputs such as mass spectrometry data and epitope maps as constraints, and has excellent single sequence prediction ability. The AlphaFold 3 (AF3), Boltz-1, and Chai-1 deep learning co-folding models show significant differences in architecture design, prediction accuracy, and physical rationality. AF3 replaces Evoformer with Pairformer to weaken the dependence on MSA, and uses atomic level attention mechanism to uniformly process protein, nucleic acid, small molecule, and post-translational modified complexes for prediction (Fromm et al., 2026). The FoldBench benchmark test showed the best performance in antibody antigen docking, with a DockQ success rate of 47.9% and a high-quality docking success rate of 13.4%, significantly ahead of other models (Xu et al., 2025). However, Masters et al.'s adversarial experiments revealed that AF3 still suffers from training data memory bias: when the binding site residue mutates to glycine or phenylalanine, the model still tends to place the ligand in the original pocket, accompanied by significant spatial conflicts. Funnel metadynamics free energy calculations confirmed that these predictions are thermodynamically unstable, indicating that they rely more on sequence pattern recognition rather than physical interaction understanding (Masters et al., 2025). As an open-source reproduction of AF3, Boltz-1 introduces pocket condition inference to optimize ligand placement, and subsequently launches the Boltz-2 version (Passaro et al., 2025). However, in the antibody antigen task, the success rate of Boltz-1 is only 21% (Gaudreault et al., 2025). Chai-1 adopts ESM embedding and MSA dual-mode input, supporting fast inference without MSA (about 10 min per antibody per A100) (Agamia and Zacharias, 2026). Overall, AF3 leads in structural accuracy but is limited by closed ecology and physical fragility, Boltz-1 excels in open source convenience but has obvious shortcomings in antibody prediction and physical effectiveness, and Chai-1 has unique value in engineering applications due to its speed advantage and constraint guidance function. All three need to be coupled with physical methods such as molecular dynamics or free energy perturbation to compensate for the inherent deficiencies of deep learning in physical understanding (Gaudreault et al., 2025; van Pinxteren and Jespers, 2025) (Table 1).

In addition, a series of specialized AI tools are forming a complementary technological ecosystem for different stages of drug development: MultiGATE (Miao et al., 2025) utilizes a two-level graph attention auto-encoder to integrate the multi-modality and spatial information in spatial multi-omics data.; AlphaGenome (Avsec et al., 2026) is capable of predicting functional genomic trajectories in different modes with single base resolution, outperforming existing models in predicting mutation effects, and enabling comprehensive genomic analysis. These tools collectively drive drug development towards a more efficient and rational direction.

### 4.2 Activity based screening: from drug library through high-throughput experimental screening, discovery of active molecules, and proposal of hypotheses to further verification

This pathway does not rely on clinical data, but on large-scale experiments to achieve a transition from a “laboratory guided clinical” model. This process requires the establishment of a ‘compound library’ containing thousands of approved drugs. For new targets or disease models, perform “high-throughput screening” in the laboratory to quickly test whether all drugs are active against the new target, and identify the “hit” drugs to resist the outbreak of the epidemic.

In addition to the protein structure prediction and virtual binding AI mentioned above, AI also plays a significant role in accelerating drug development in high-throughput screening. DrugCLIP has shown great advantages in protein ligand ultra high-throughput virtual screening, as it can quantify protein pockets and small molecules. The screening speed is millions of times faster than traditional methods, and the daily processing capacity reaches 31 trillion times (Jia et al., 2026). The Massachusetts Institute of Technology and Tufts University jointly proposed the ConPLex platform, which is based on a large language model and can match proteins and drug molecules without calculating 3D structures, with extremely fast screening speed (Singh et al., 2023). PLANET (TaigetMol) is based on graph neural network for affinity prediction, using target 3D structure and ligand 2D structure. It only took less than 1% of Glide’s computation time to complete the same task (Zhang et al., 2024).

In terms of data analysis and integration, CLIPn uses deep contrastive learning to align high-throughput screening numbers from different sources, achieve cross dataset transitivity prediction, build a “universal language” for data, and improve the accuracy of predicting unknown drug functions (Bao et al., 2025). Avatar Discovery, published by Mavatar in Sweden (The company website is: www.avatar.com), is based on the Deep Integrated Network Analysis (DiNA) framework, helping researchers quickly analyze complex biological data and gain disease insights.

AI can also accelerate pre drug ADMET property prediction (Gupta et al., 2021). Artificial intelligence driven ADMET prediction has moved from traditional quantitative structure-activity relationship (QSAR) models to the era of deep learning, achieving a paradigm shift from molecular representation learning to multi task collaborative prediction. Due to the subjectivity and insufficient cross task generalization ability of traditional learning methods in feature engineering, deep learning models use graph neural networks (GNNs), Transformer architectures, and pre trained language models (such as ChemBERTa, MolGPT) to automatically extract molecular topology and semantic features, significantly improving prediction accuracy and data efficiency; Especially, multi task learning frameworks effectively alleviate the problem of data sparsity by capturing the intrinsic correlations between ADMET attributes through shared representation layers, while transfer learning and domain adaptation strategies further enhance the model’s extrapolation ability in new targets and new chemical spaces (Malarvannan et al., 2026). The current integrated AI platform has achieved full process coverage from early screening of drug properties to toxicity mechanism analysis: deep neural networks based on large-scale metabolomics data can predict Drug-drug interactions (DDIs) and organ specific toxicity, and hybrid intelligent systems combined with physiological based pharmacokinetics (PBPK) models can dynamically simulate *in vivo* Exposure-Response relationships, providing quantitative basis for precise drug delivery (Lee et al., 2022). However, the “black box” nature of deep learning models and the bias in chemical spatial coverage remain the core bottlenecks that constrain their regulatory recognition. In the future, it is necessary to integrate prior knowledge of physics and chemistry to construct interpretable AI frameworks, and establish a cross laboratory standardized validation system to promote the transformation of ADMET predictions from research tools to decision basis.

### 4.3 Mechanism based reasoning: from biological knowledge/mechanism research through logical reasoning to propose hypotheses to laboratory verification

Network medicine emphasizes the complete pathway of multi gene interaction network through disease mechanism to treatment intervention, and has practical biological significance. Albert-L ászló Barabási pioneered this concept in 2011 and systematically elucidated how to apply complex network theory to human disease research (Barabási et al., 2011). It relies on a specific set of analysis methods and data resources. In terms of drug repositioning, its advantage lies in analyzing the comorbidity network of diseases and the location of drug targets in the network, in order to find the possibility of using known drugs to treat other diseases (Ivanov, 2021). The core lies in “understanding” and “searching” - understanding the mechanisms of two or more diseases, understanding the mechanisms of drug action, and then finding a reasonable connection between the two.

Especially in the sharing of pathways/targets, utilization of side effects, and key upstream and downstream regulation, it plays an important role. However, Arash Sadri traced back to drug research and development and proposed that only 9.4% of small molecule drugs were discovered through “target-based” assays, indicating that the research efficiency of this method is not optimistic (Sadri, 2023).

Off target effects of drugs refer to the phenomenon where drugs interact with biomolecules outside the expected target, leading to unexpected pharmacological or toxic reactions. The combination of drug target recognition, early affinity chromatography based target recognition, transcriptome range compound profiling analysis, chemical genomics, and yeast two hybrid methods were replaced by chemical proteomics due to high false positive rates and limited applicability (Zou et al., 2024). Currently, the deep integration of artificial intelligence and chemical proteomics is driving off target effects into the era of “prediction pre validation”. As mentioned earlier, AI can screen and predict the binding probability of multiple potential targets in large quantities, and verify the ADMET property prediction of drugs, assisting in the basic analysis of drugs. However, due to the “black box” problem of AI, drug development still cannot be separated from specific experimental verification, and the closed-loop era of “prediction verification optimization” of off target drug effects still poses certain challenges.

In terms of experimental verification, microfluidic organ chips have significant advantages in predicting human drug efficacy and toxicity (Ingber, 2022). Satta et al. summarized the mechanism of immune endothelial interaction injury after SARS-CoV-2 infection reproduced using vascularized lung chips (Satta et al., 2023). However, despite significant advancements in materials and technological processes, the single organ model is difficult to simulate the complex physiological and systemic pathological processes of the human body. The developmental changes, aging, and chronic disease dynamics of physiological organs cannot be simulated, resulting in insufficient clinical application and translational capabilities.

## 5 Challenges

Despite the promising prospects of AI driven drug repositioning, its development still faces multiple challenges. Data quality is the primary bottleneck, as the heterogeneity, incompleteness, and systematic bias of biomedical data directly affect model performance. Data from different experimental platforms and using different labeling standards are like a difficult “language” to communicate, Meanwhile, Alice B Popejoy mentioned that 96% of participants in genome-wide association studies (GWAS) are of European descent (Popejoy and Fullerton, 2016), so missing data for specific populations may lead to prediction bias.

The widely used deep learning models currently have a “black box” characteristic, making it difficult to trace decision logic and verify the rigor of prediction results and reasoning processes (Aggarwal et al., 2022). Gunning et al. pointed out as early as 2019 that interpretability is a necessary prerequisite for users to effectively understand, trust, and manage powerful AI applications (Gunning et al., 2019). When faced with multiple AI prediction results that are different, the lack of transparency and interpretability seriously hinders AI recommended drugs from entering clinical trials and practical applications. AI + assistance in pharmacology should align with the FDA’s GMLP and India’s Responsible AI Principles (Suryadevara et al., 2026),Transitioning to explainable artificial intelligence (XAI) is essential. Khalili et al. focused on the field of epidemiology and pointed out that XAI has irreplaceable value in enhancing policymakers’ trust in AI models and optimizing non pharmacological intervention strategies in public health crises such as SARS-CoV-2 (Khalili and Wimmer, 2024). Qadri et al. systematically reviewed the empowering mechanisms of XAI in target recognition, molecular property prediction, ADME evaluation, and clinical trial design, emphasizing that XAI effectively bridges the gap between computational prediction and pharmaceutical practice by elucidating model decision logic (Qadri et al., 2025). However, although XAI provides actionable insights for scientific discovery, concerns regarding its reliability and robustness remain (Wu et al., 2023). Furthermore, systematic reviews across healthcare AI domains reveal persistent methodological heterogeneity and insufficient effectiveness evaluation (Caterson et al., 2024). Hettikankanamage et al.'s cross disciplinary systematic review found that Shapley Additive eXPlanations (SHAP) appeared with a frequency of up to 79.5% in 44 quantitative prediction studies. However, its additive and causal assumptions have theoretical limitations in heterogeneous biomedical data, and current research generally lacks structured human subject usability validation, constituting a key gap in clinical translation (Hettikankanamage et al., 2025).

At the same time, the clinical translation gap remains significant, and there is a lack of efficient and standardized connection platforms between computational prediction and wet experimental validation, resulting in a large number of potential AI predictions not being able to enter the subsequent experimental validation stage. The complexity of biological systems creates a gap between *in vitro* experimental results and actual *in vivo* efficacy, further increasing the difficulty of translating predictions into clinical practice.

## 6 Conclusion

Viruses often exhibit structural changes, which may be the cause of pandemics (Handa et al., 2025). AI in predicting real-time viral mutations and adjusting repurposed drug. It is very important to deal with outbreaks of infectious diseases, and real-time prediction or even advance prediction of virus mutations is a major challenge. Most models such as ViralForesig (Liu et al., 2025) and EVEscape (Thadani et al., 2023) focus on sequence statistical operations, without crossing the multi-scale gap of “from sequence, to conformation, to function, to popularity. Under the prediction of the “black box” model, there is great room for improvement in its influence on policymakers and drug managers such as the FDA.

Collaboration across communities is a key approach to driving AI driven virus mutation prediction and drug repositioning from theory to practice. Each model has its own advantages. Establishing a unified standard and integrating the complementary advantages of different methodological systems to break through the inherent limitations of a single paradigm will greatly promote the progress of AI + drug repositioning research.
